# Supplementary material for: α-Trifluoromethyl Chalcones as Potent Anticancer Agents for Androgen Receptor-Independent Prostate Cancer
Source: Molecules. 2021 May 10;26(9):2812. doi: 10.3390/molecules26092812 (PMC8126091; doi:10.3390/molecules26092812)
Supplement: Supplementary file 1 [file molecules-26-02812-s001.zip › molecules-1208175-supplementary.pdf]

Supporting Information

α-Trifluoromethyl Chalcones as Potent Anticancer Agents for Androgen Receptor-Independent Prostate Cancer

Yohei Saito,[†] *Atsushi Mizokami,* [*,‡] *Kouji Izumi,*[‡] *Renato Naito,*[‡] *Masuo Goto*[§] *and Kyoko Nakagawa-Goto*[*,†,§]

[†]School of Pharmaceutical Sciences, College of Medical, Pharmaceutical and Health Science, Kanazawa University, Kanazawa, 920-1192, Japan

[‡]Department of Integrative Cancer Therapy and Urology, School of Medical Sciences, Kanazawa University, Kanazawa, 920-1192, Japan

[§]Chemical Biology and Medicinal Chemistry, Eshelman School of Pharmacy, University of North Carolina, Chapel Hill, North Carolina 27599, United States

Table of Contents

Figures S1–S40. ^{1}H NMR, ^{13}C NMR, FT-IR, and UV-VIS spectra of compounds **2–11** S2–22

Table S1. Detailed information of detected binding proteins..............................S23

Figure S1. ^{1}H NMR spectrum of compound **2** (600 MHz, in $CDCl_3$)

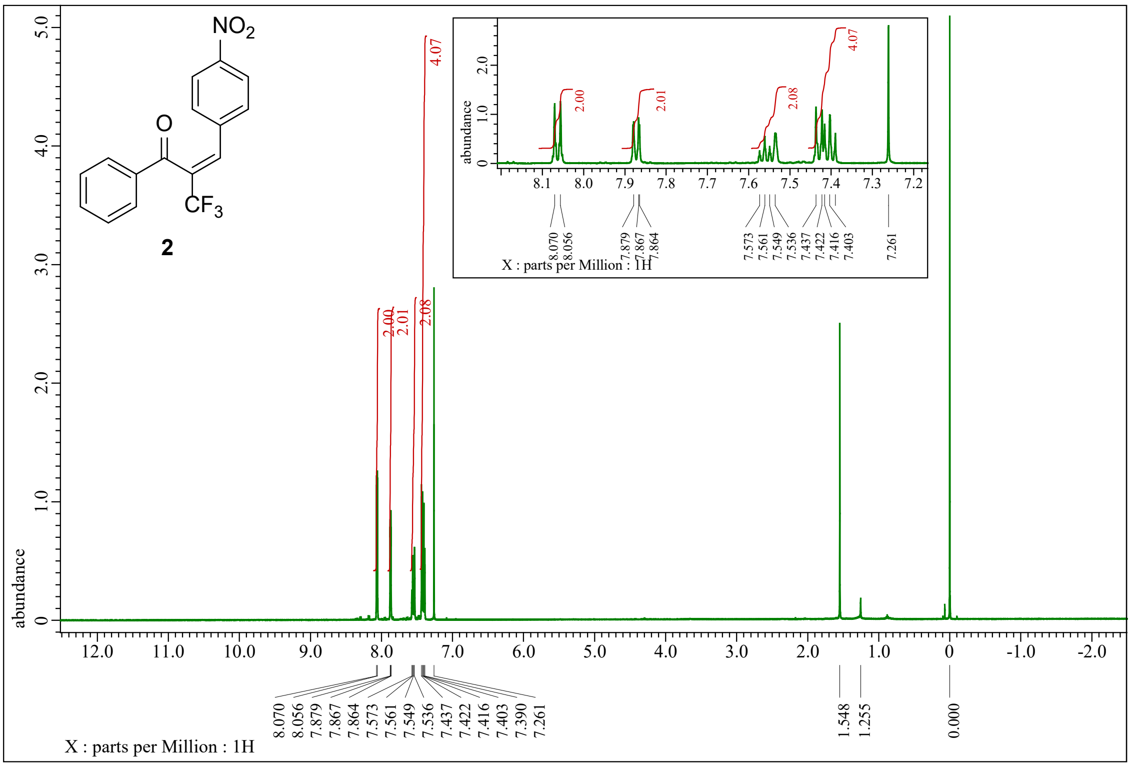

Figure S2. ^{13}C NMR spectrum of compound **2** (150 MHz, in $CDCl_3$)

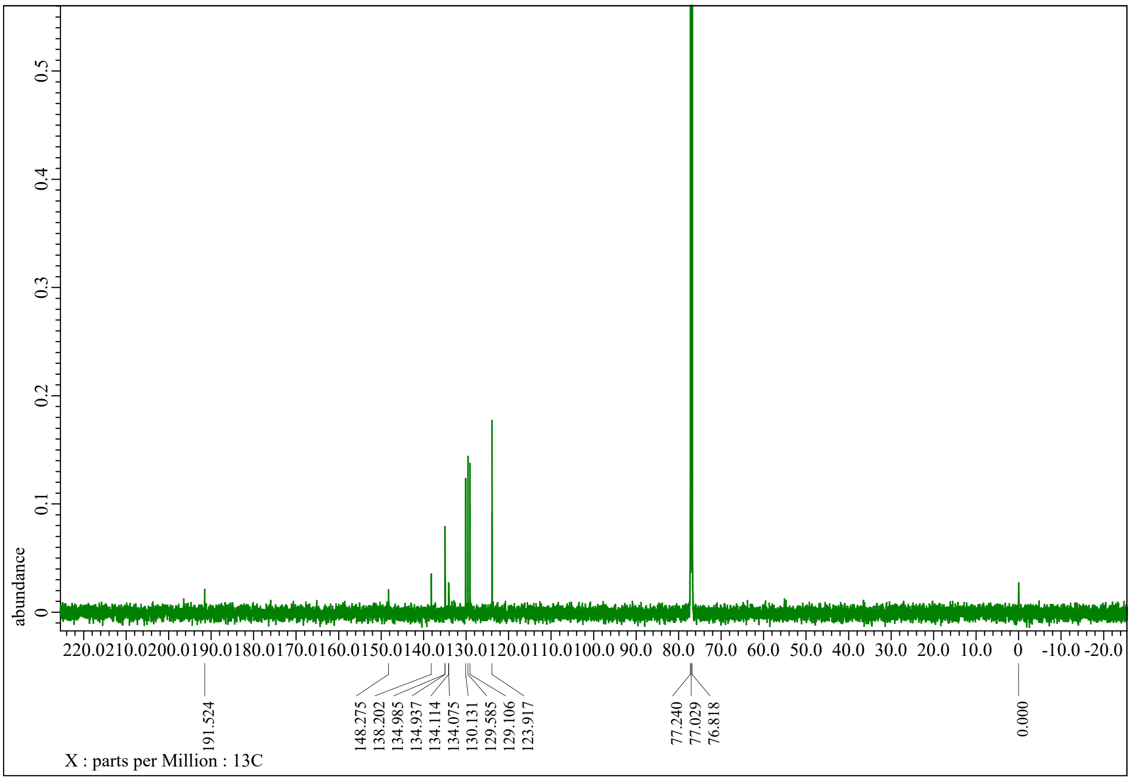

Figure S3. FT-IR spectrum of compound **2** (neat)

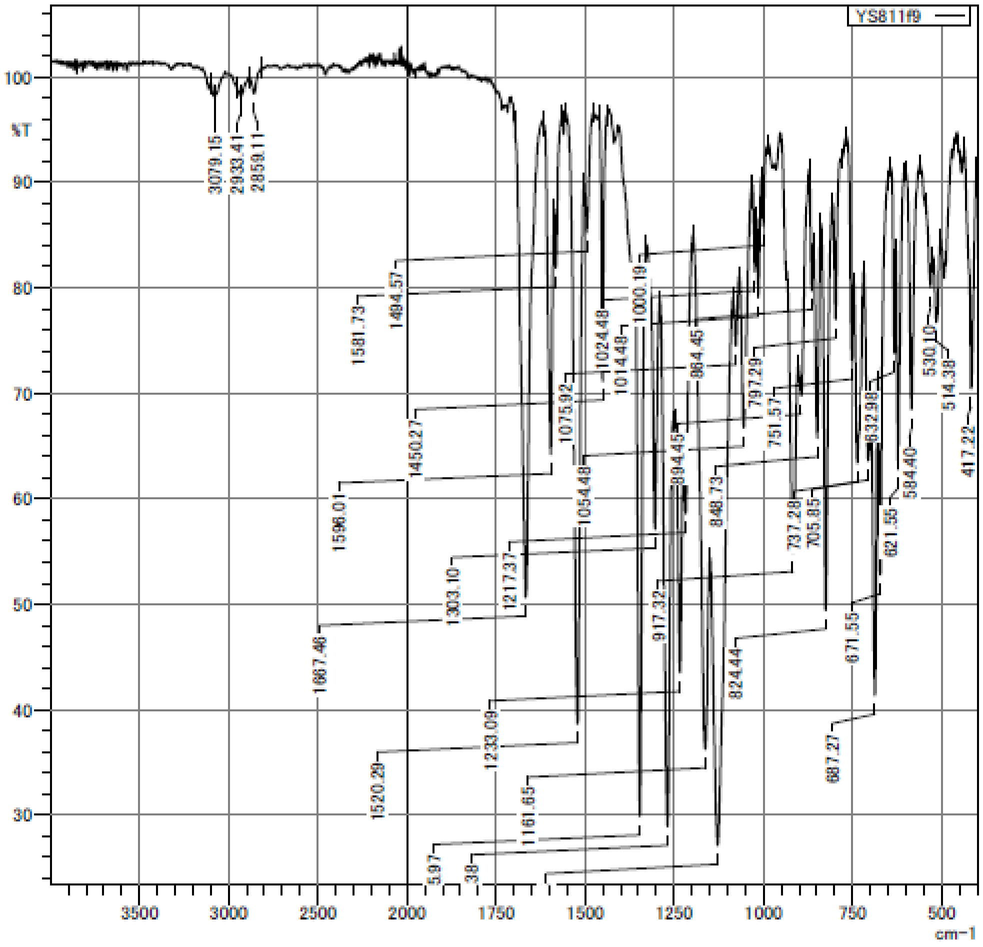

Figure S4. UV-VIS spectrum of compound **2** (MeCN/H_2O, 1:1)

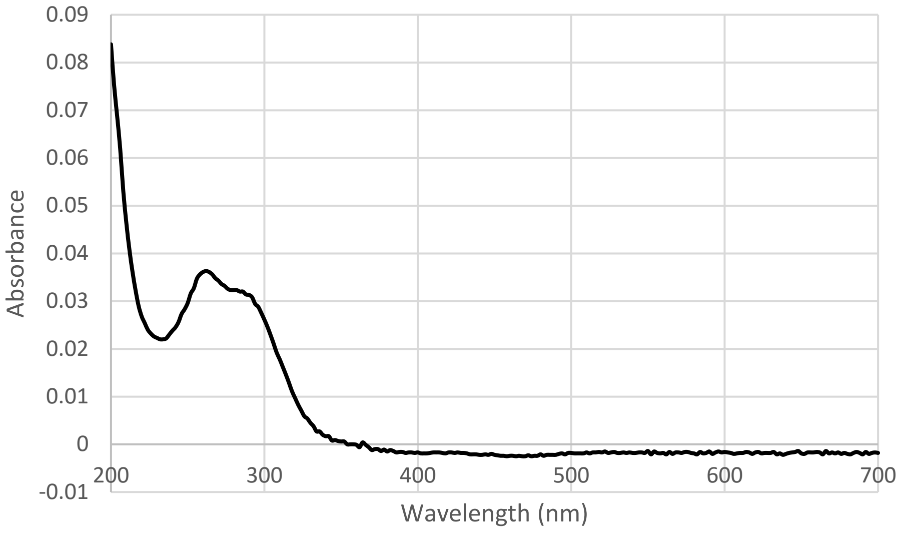

Figure S5. ^{1}H NMR spectrum of compound **3** (400 MHz, in $CDCl_3$)

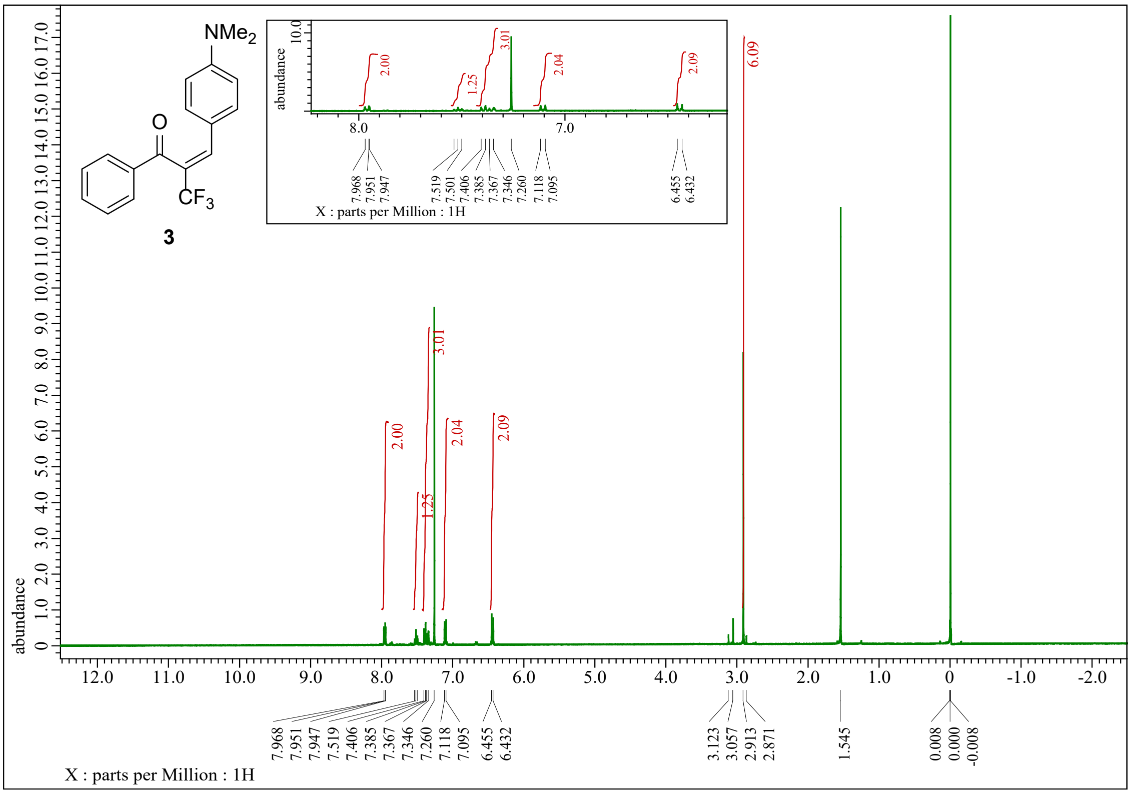

Figure S6. ^{13}C NMR spectrum of compound **3** (100 MHz, in $CDCl_3$)

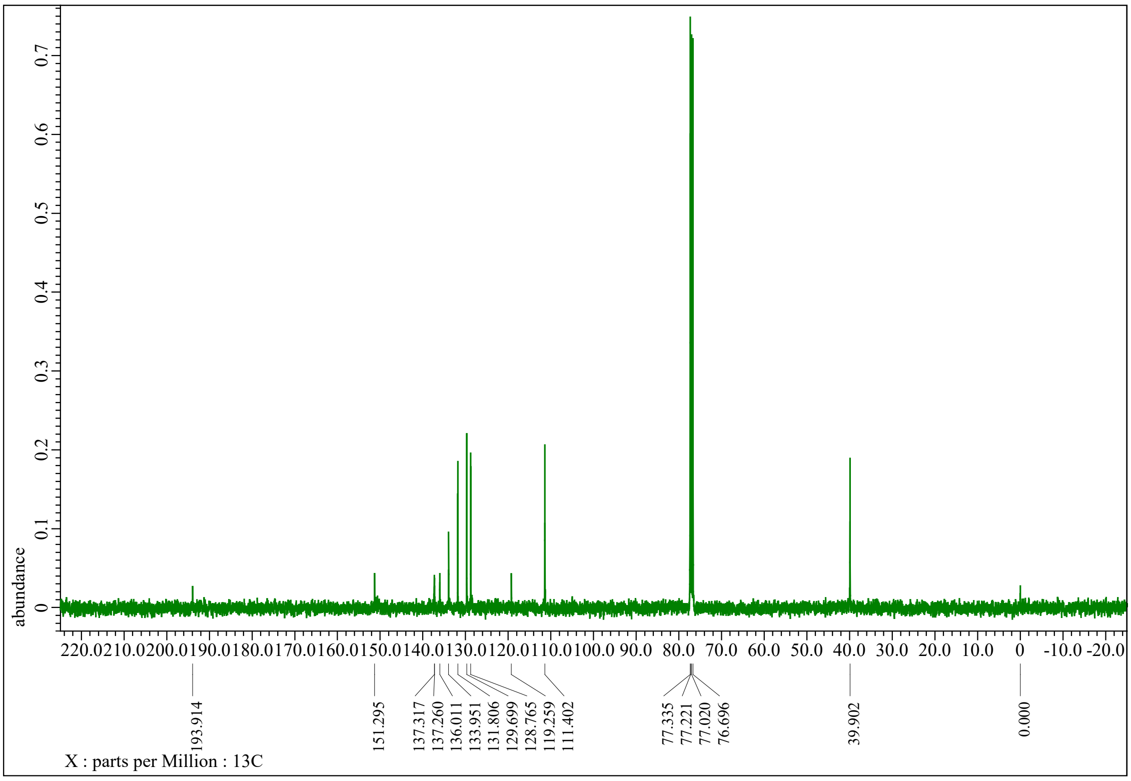

Figure S7. FT-IR spectrum of compound **3** (neat)

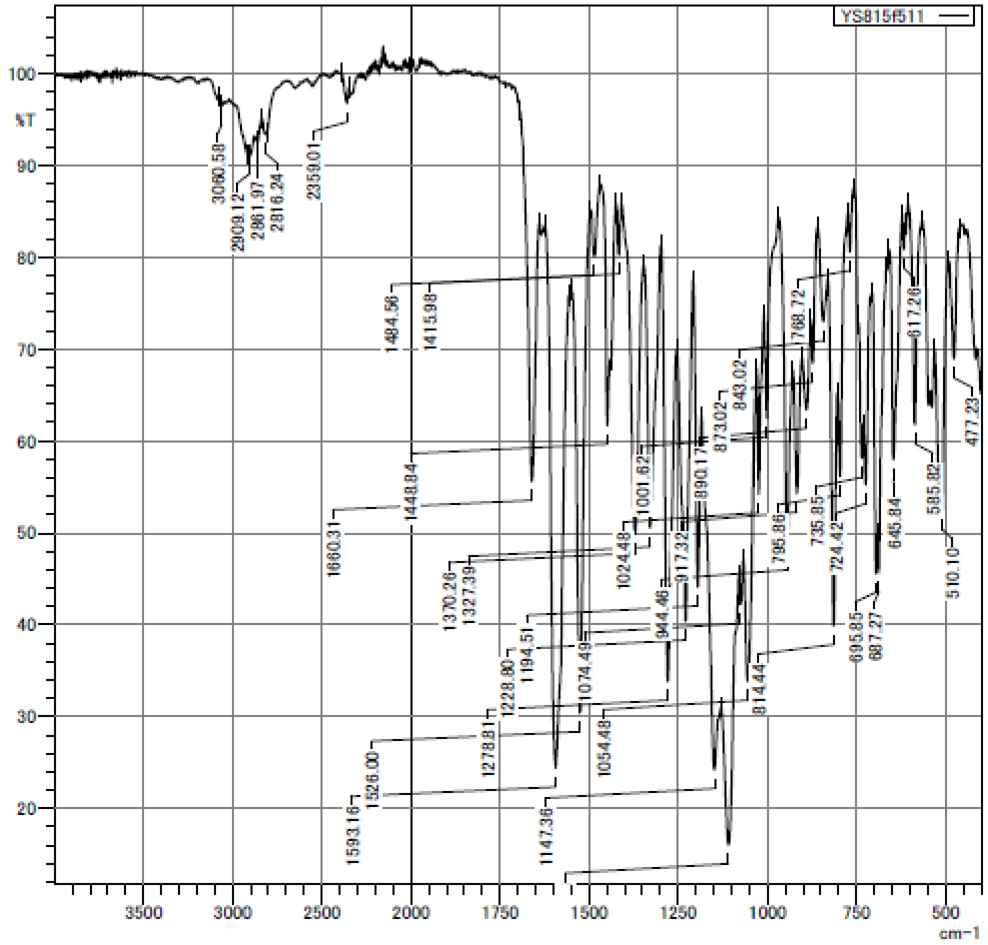

Figure S8. UV-VIS spectrum of compound **3** ($MeCN/H_2O$, 1:1)

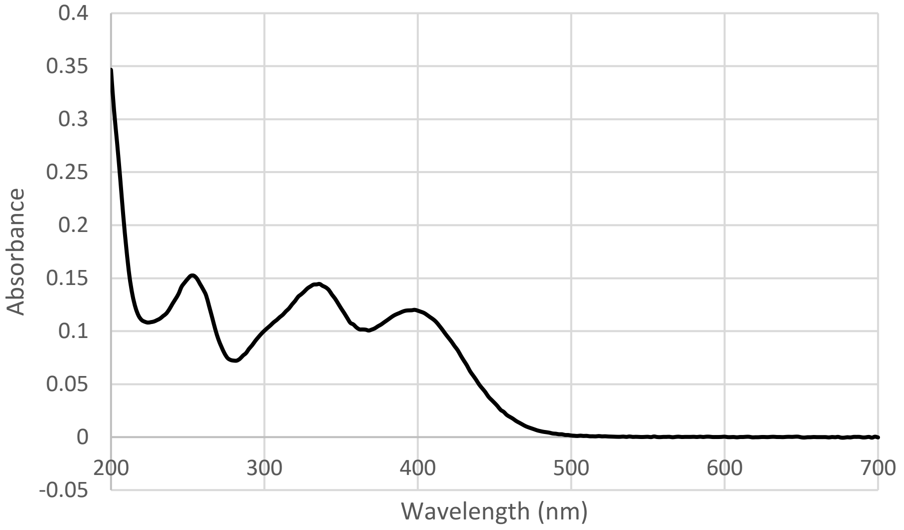

Figure S9. ^{1}H NMR spectrum of compound **4** (400 MHz, in $CDCl_3$)

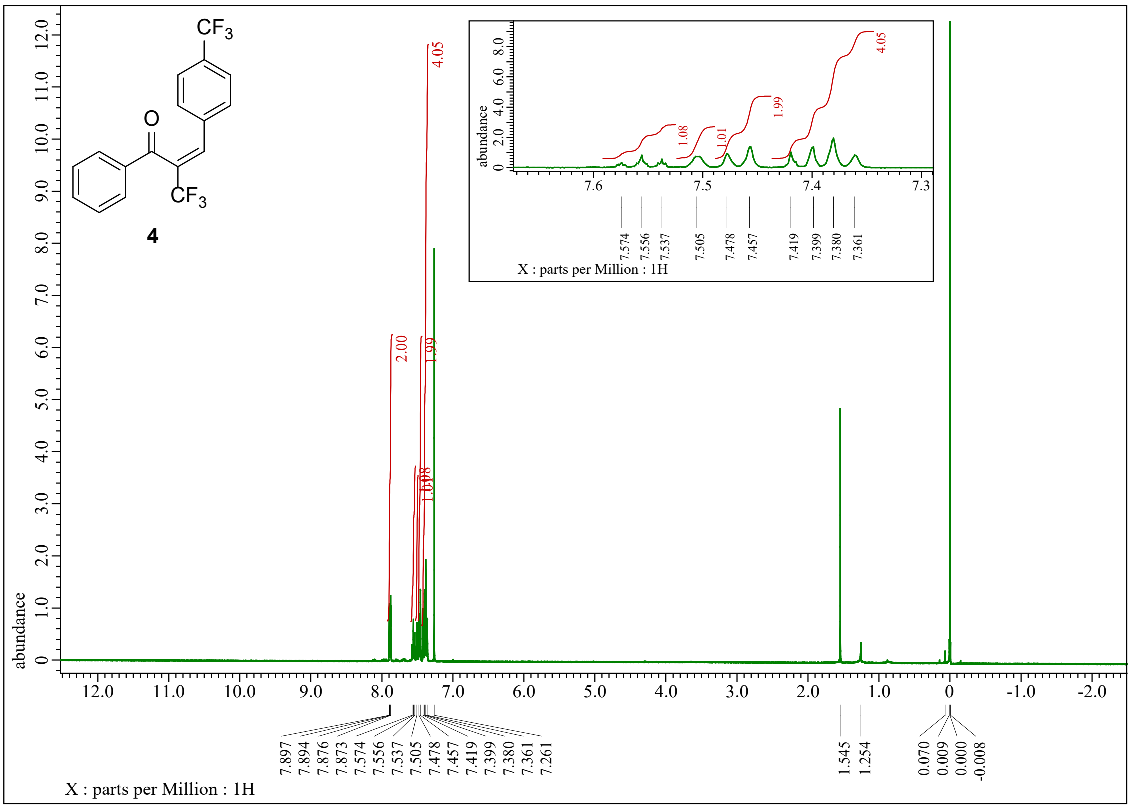

Figure S10. ^{13}C NMR spectrum of compound **4** (150 MHz, in $CDCl_3$)

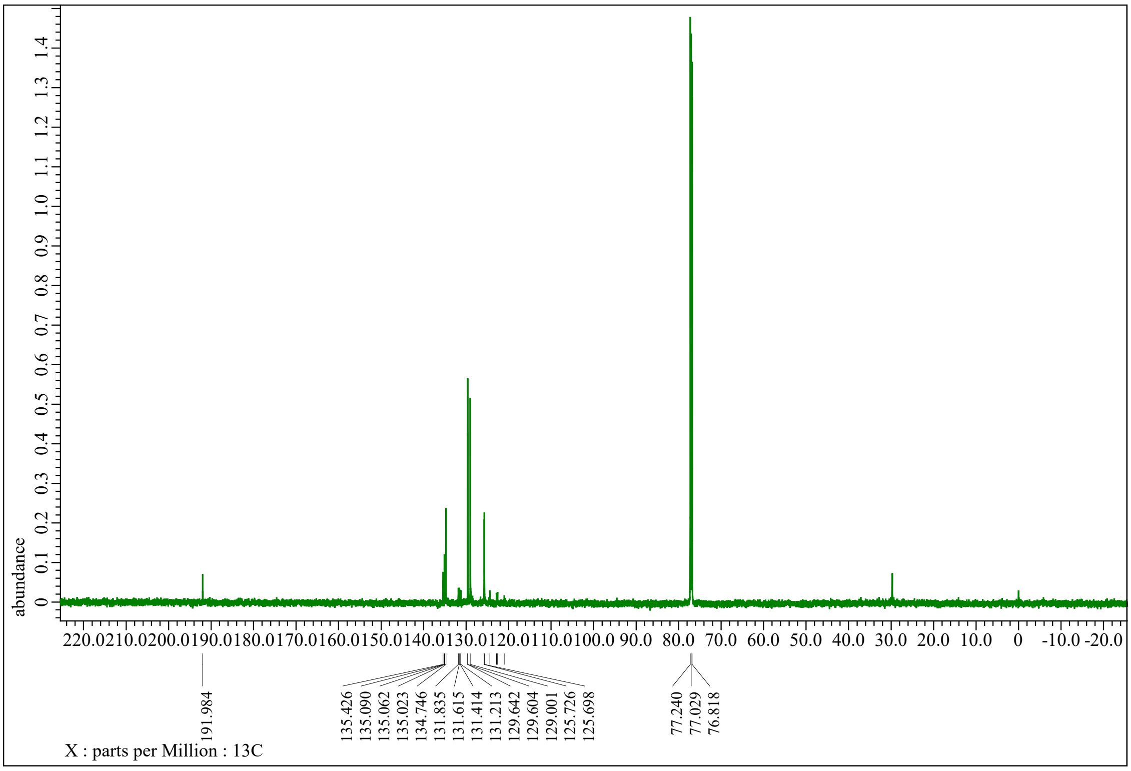

Figure S11. FT-IR spectrum of compound **4** (neat)

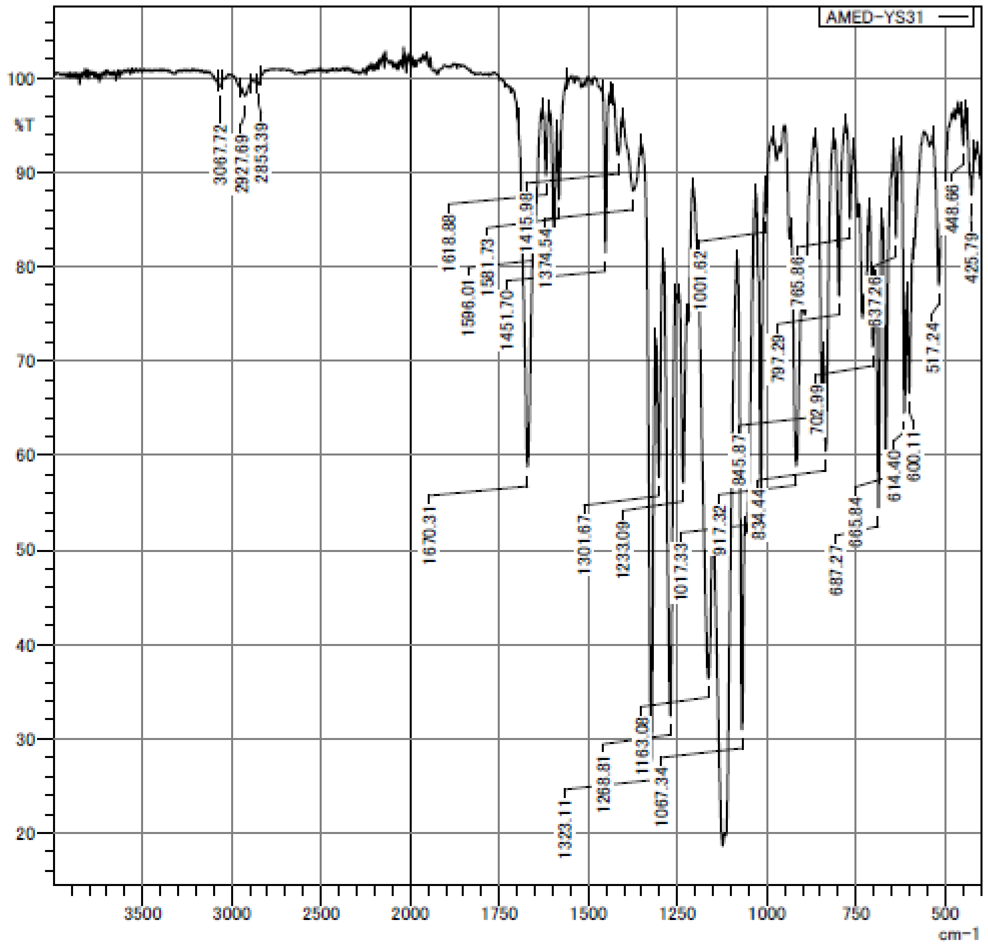

Figure S12. UV-VIS spectrum of compound **4** (MeCN/H_2O, 1:1)

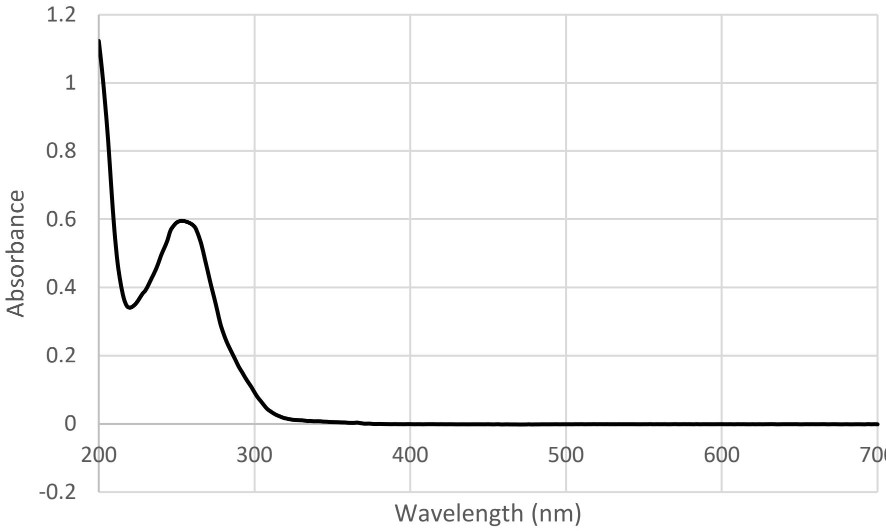

Figure S13. ^{1}H NMR spectrum of compound **5** (600 MHz, in $CDCl_3$)

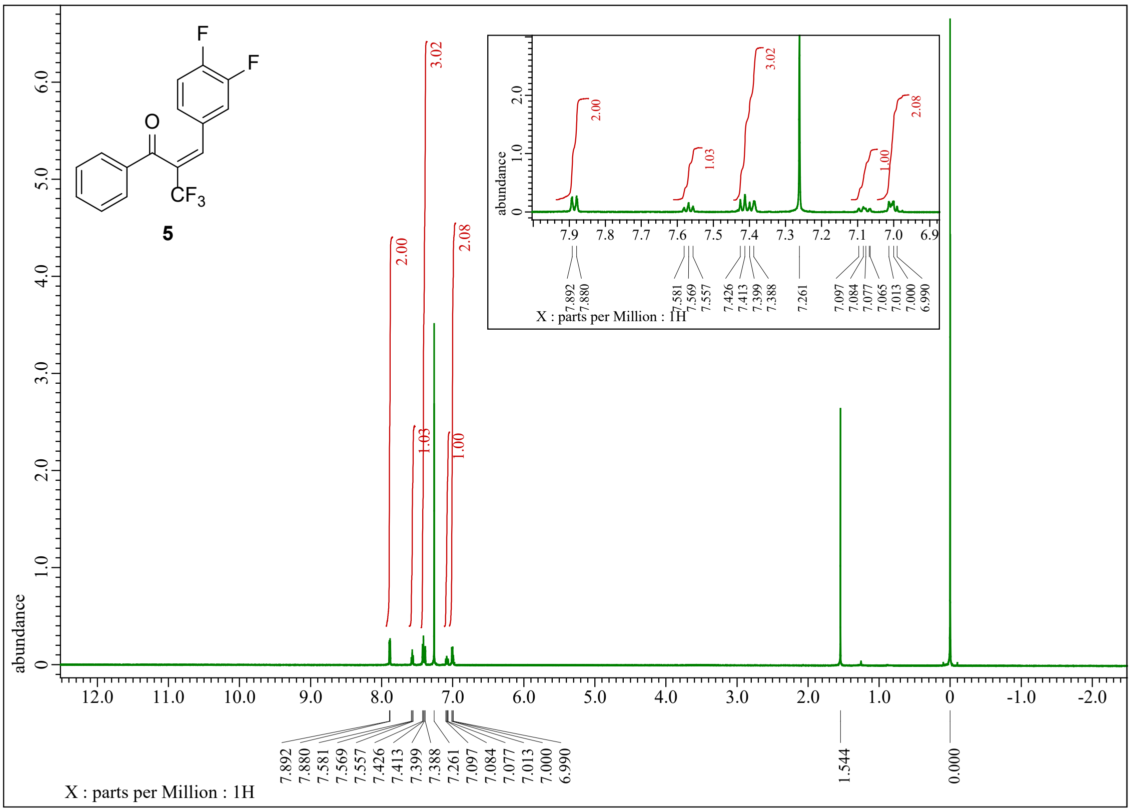

Figure S14. ^{13}C NMR spectrum of compound **5** (150 MHz, in $CDCl_3$)

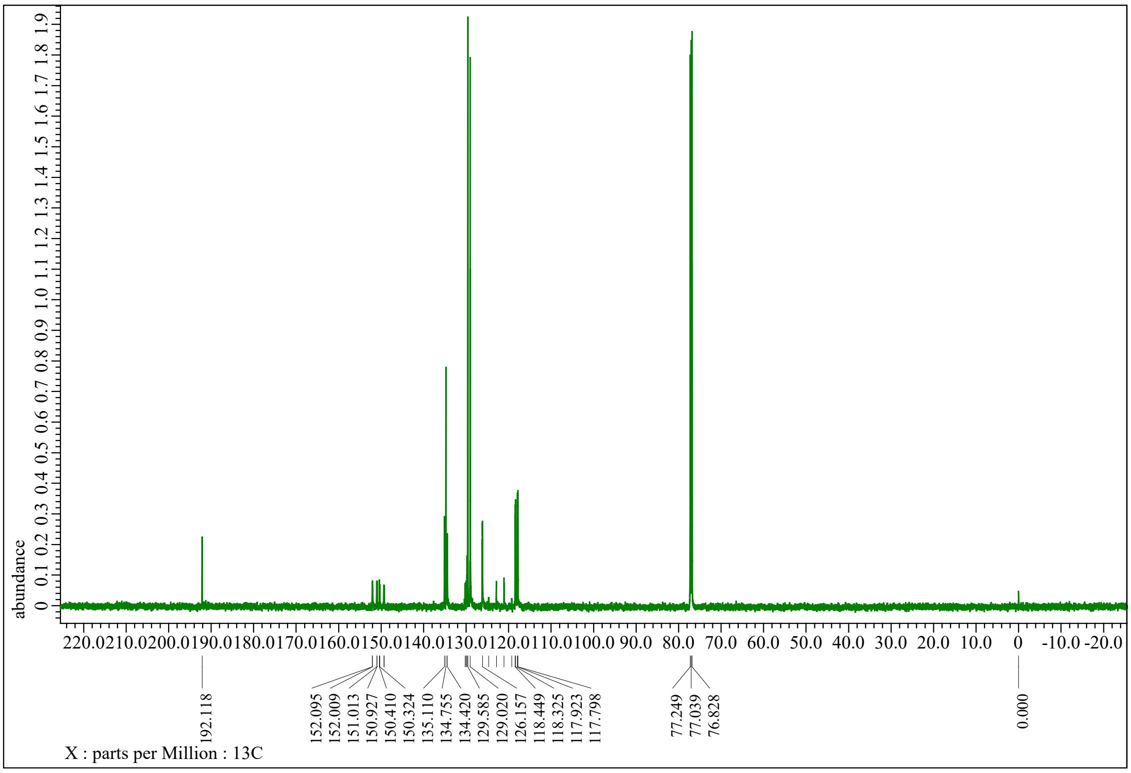

Figure S15. FT-IR spectrum of compound **5** (neat)

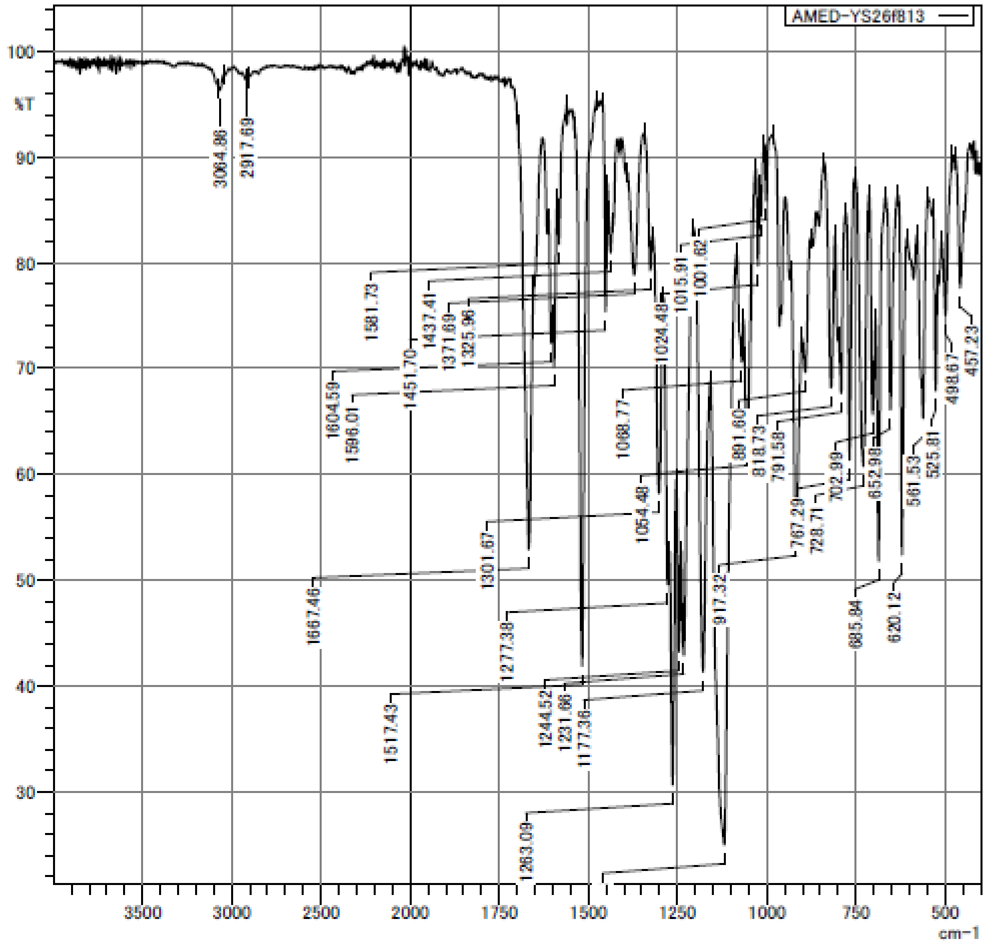

Figure S16. UV-VIS spectrum of compound **5** (MeCN/H_2O, 1:1)

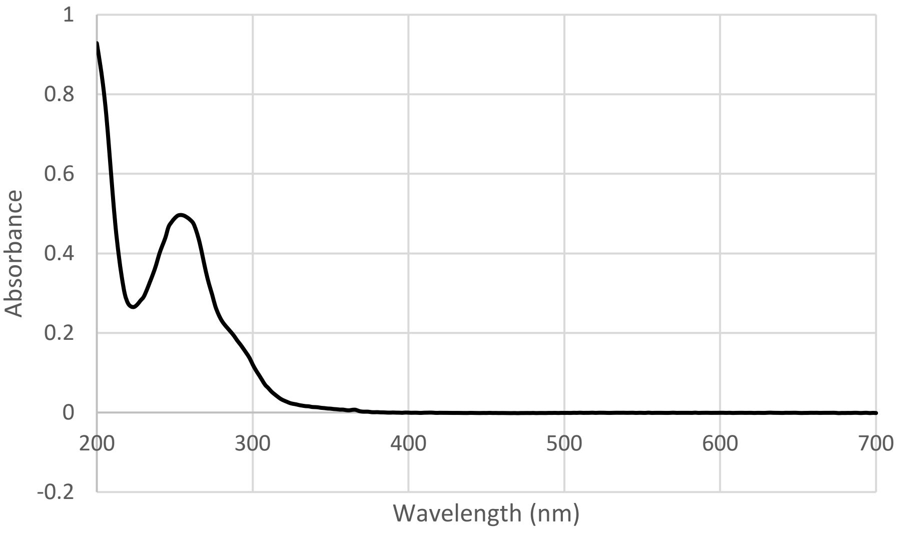

Figure S17. ^{1}H NMR spectrum of compound **6** (600 MHz, in $CDCl_3$)

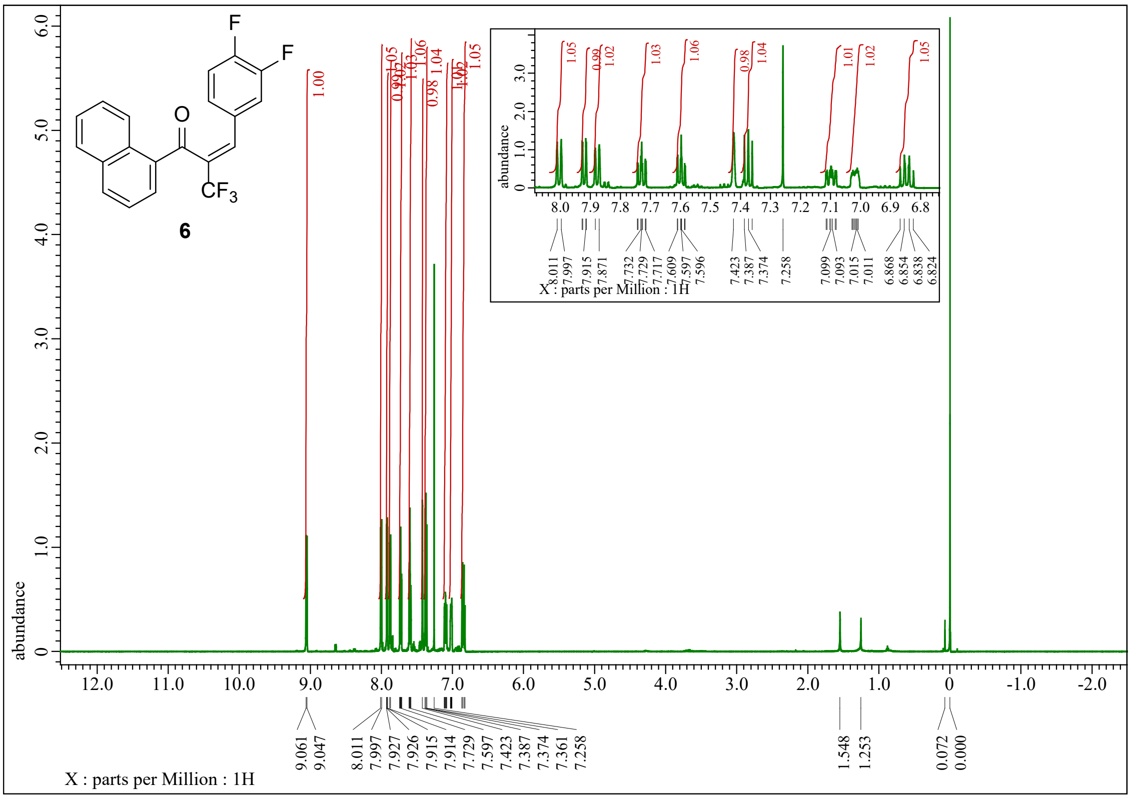

Figure S18. ^{13}C NMR spectrum of compound **6** (150 MHz, in $CDCl_3$)

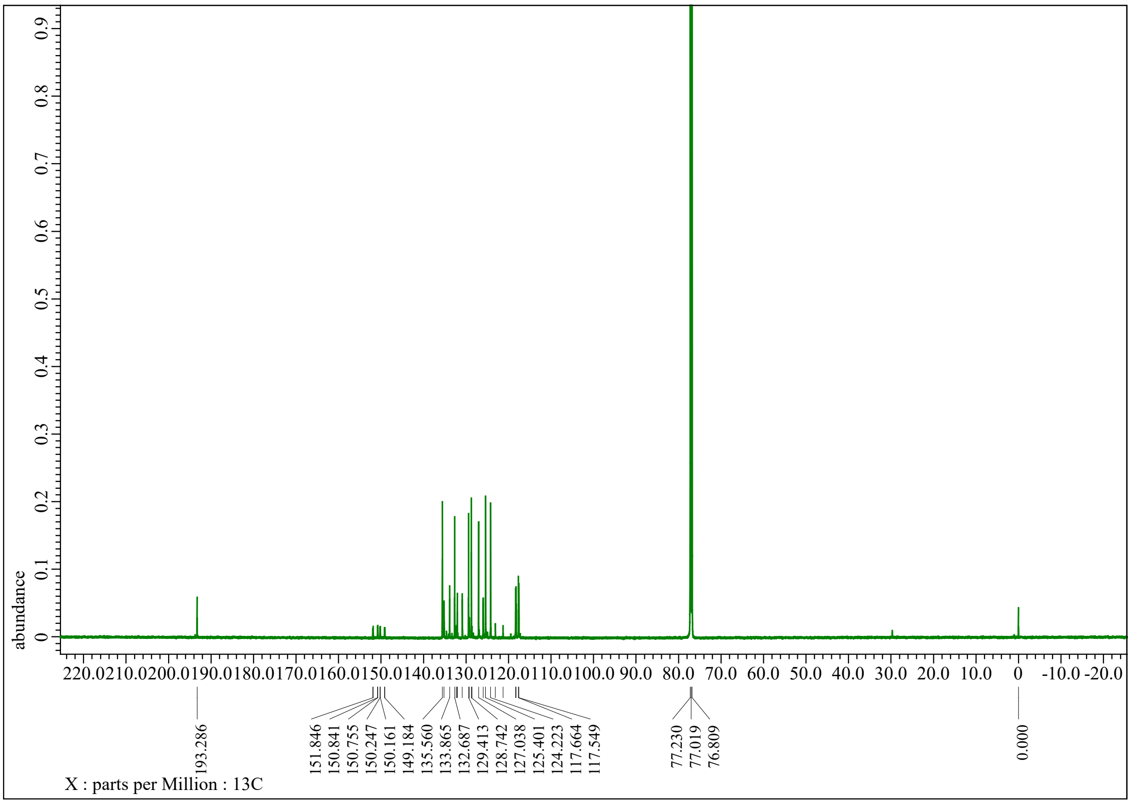

Figure S19. FT-IR spectrum of compound **6** (neat)

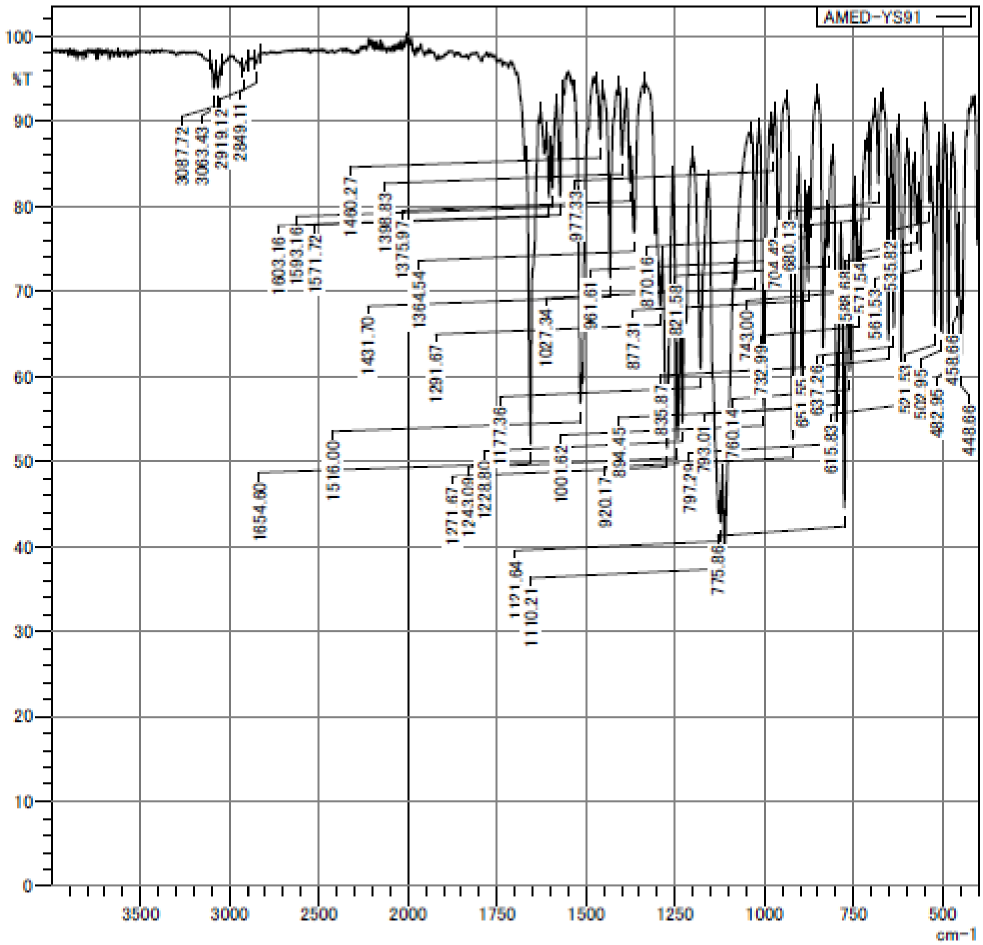

Figure S20. UV-VIS spectrum of compound **6** ($MeCN/H_2O$, 1:1)

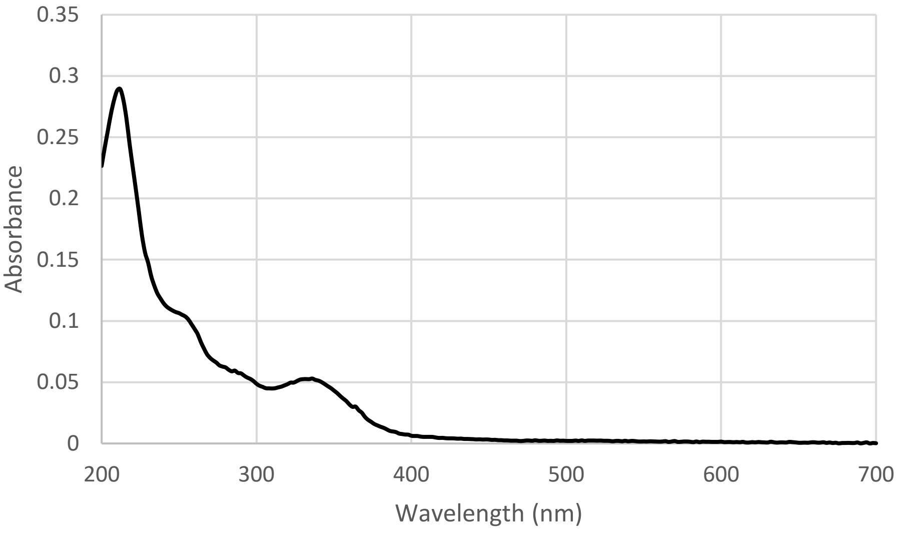

Figure S21. ^{1}H NMR spectrum of compound **7** (600 MHz, in $CDCl_3$)

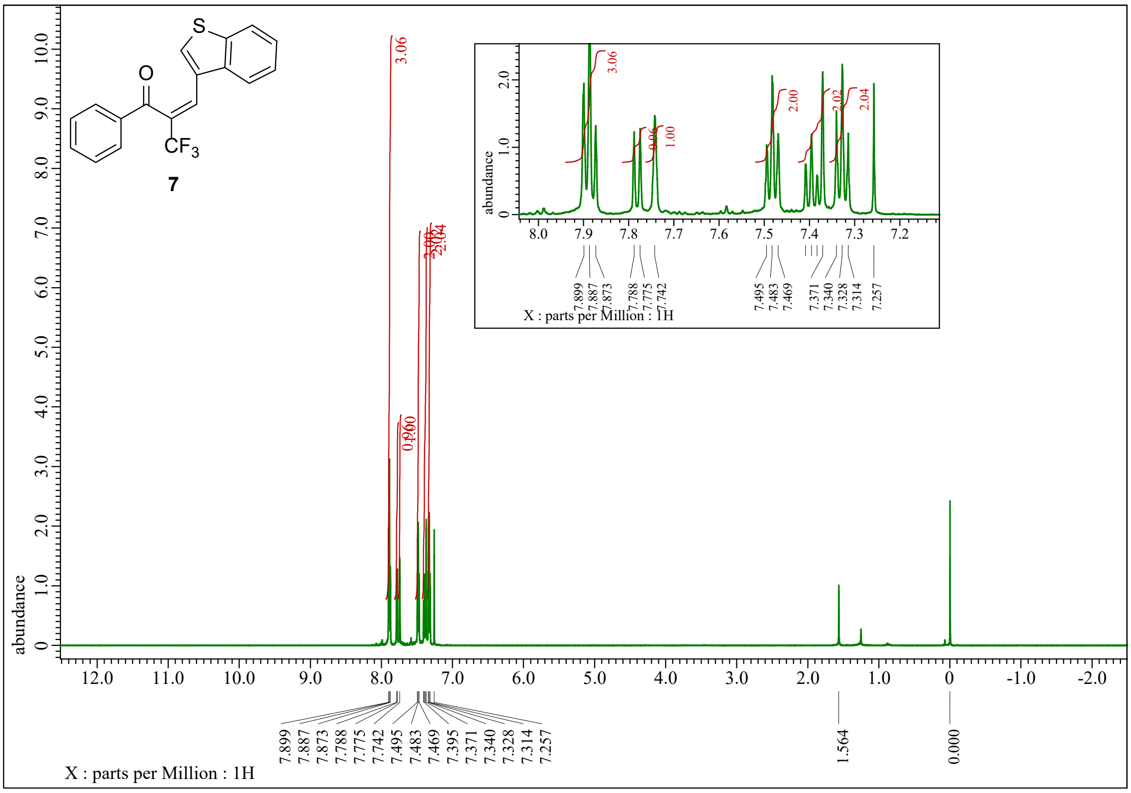

Figure S22. ^{13}C NMR spectrum of compound **7** (150 MHz, in $CDCl_3$)

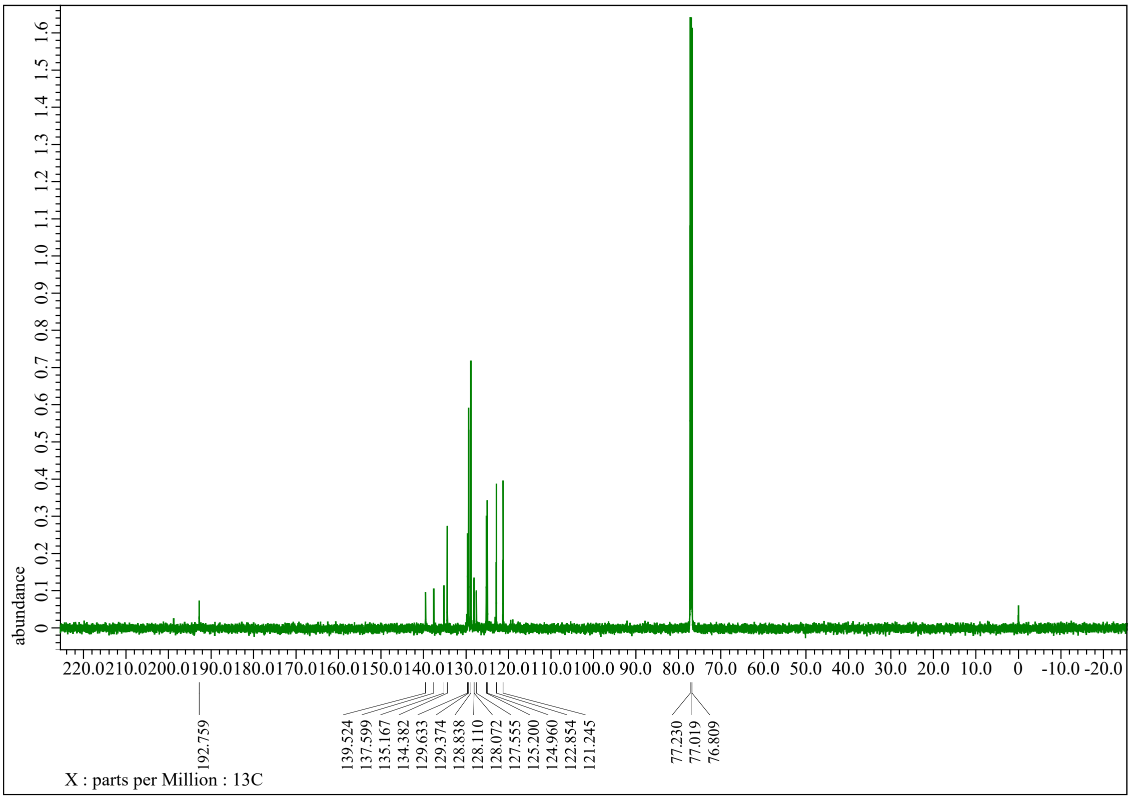

Figure S23. FT-IR spectrum of compound **7** (neat)

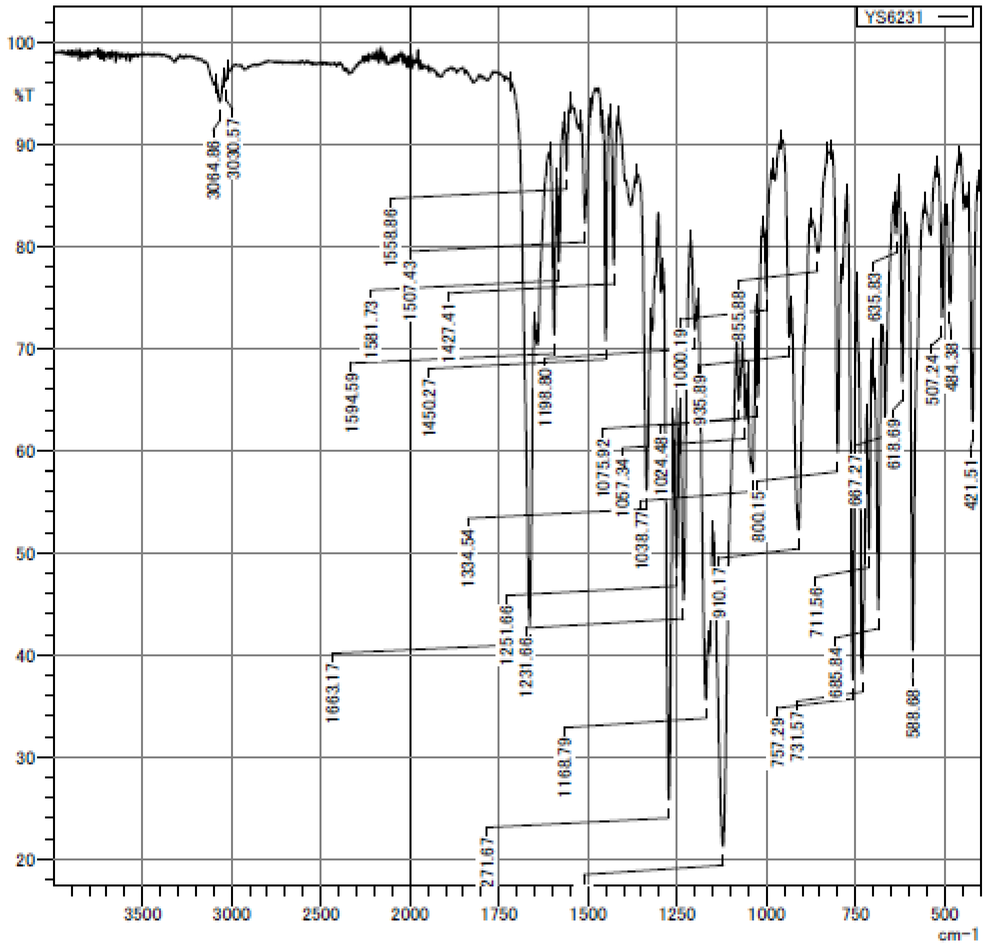

Figure S24. UV-VIS spectrum of compound **7** (MeCN/H_2O, 1:1)

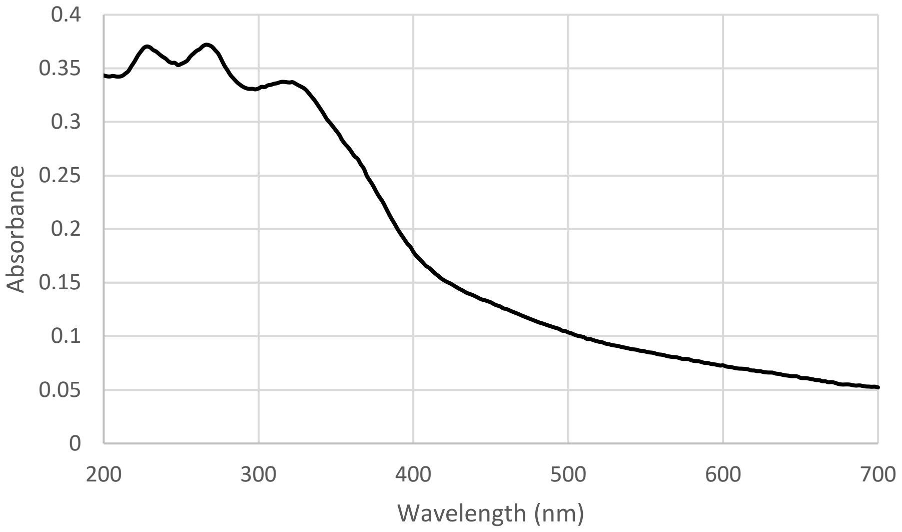

Figure S25. ^{1}H NMR spectrum of compound **8** (400 MHz, in $CDCl_3$)

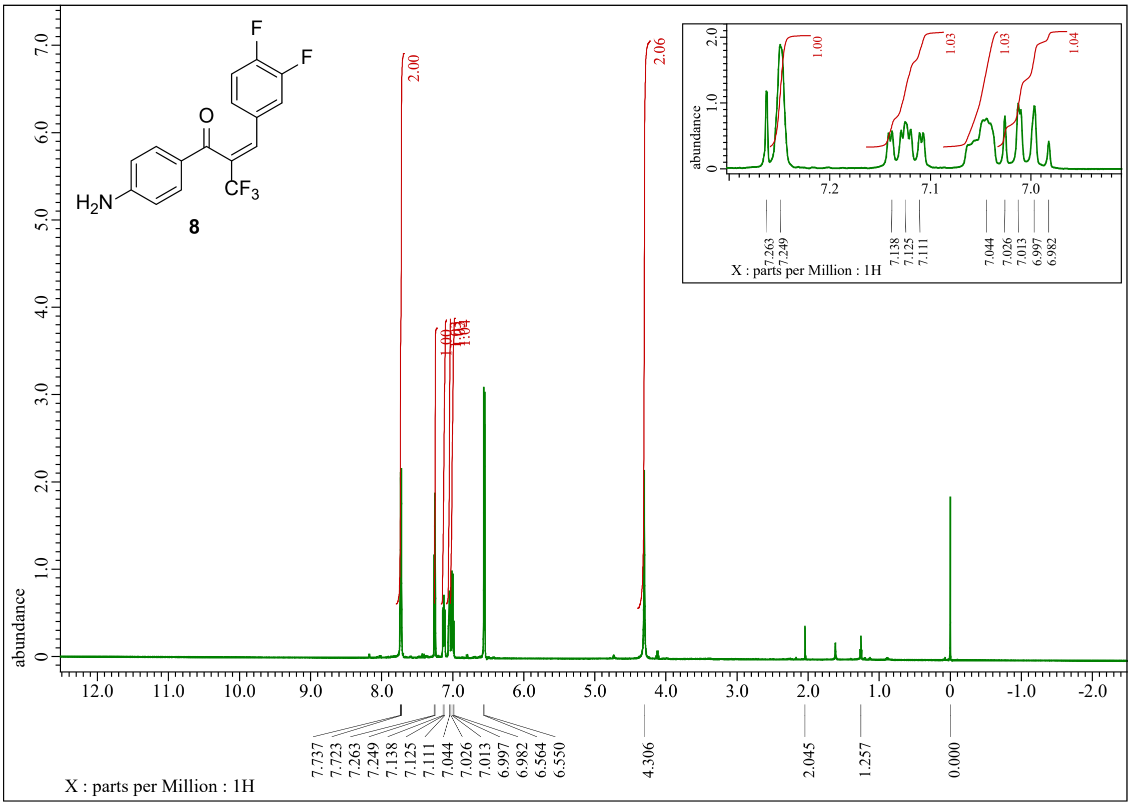

Figure S26. ^{13}C NMR spectrum of compound **8** (150 MHz, in $CDCl_3$)

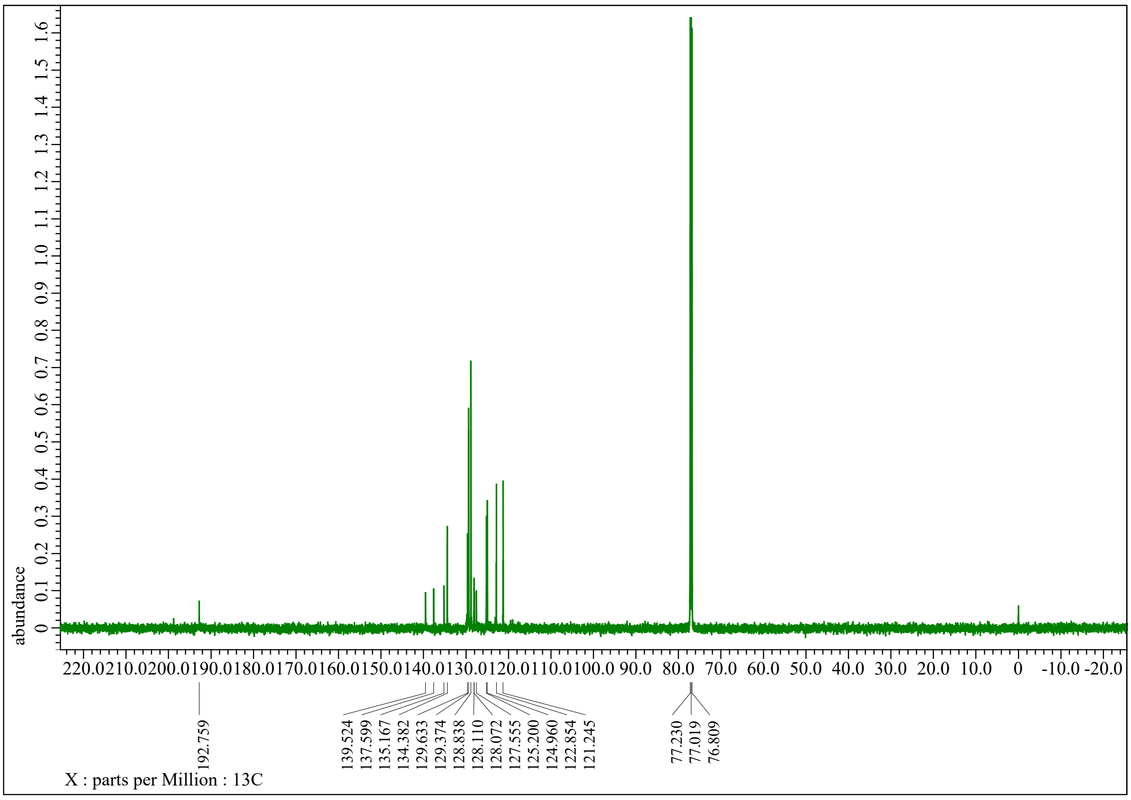

Figure S27. FT-IR spectrum of compound **8** (neat)

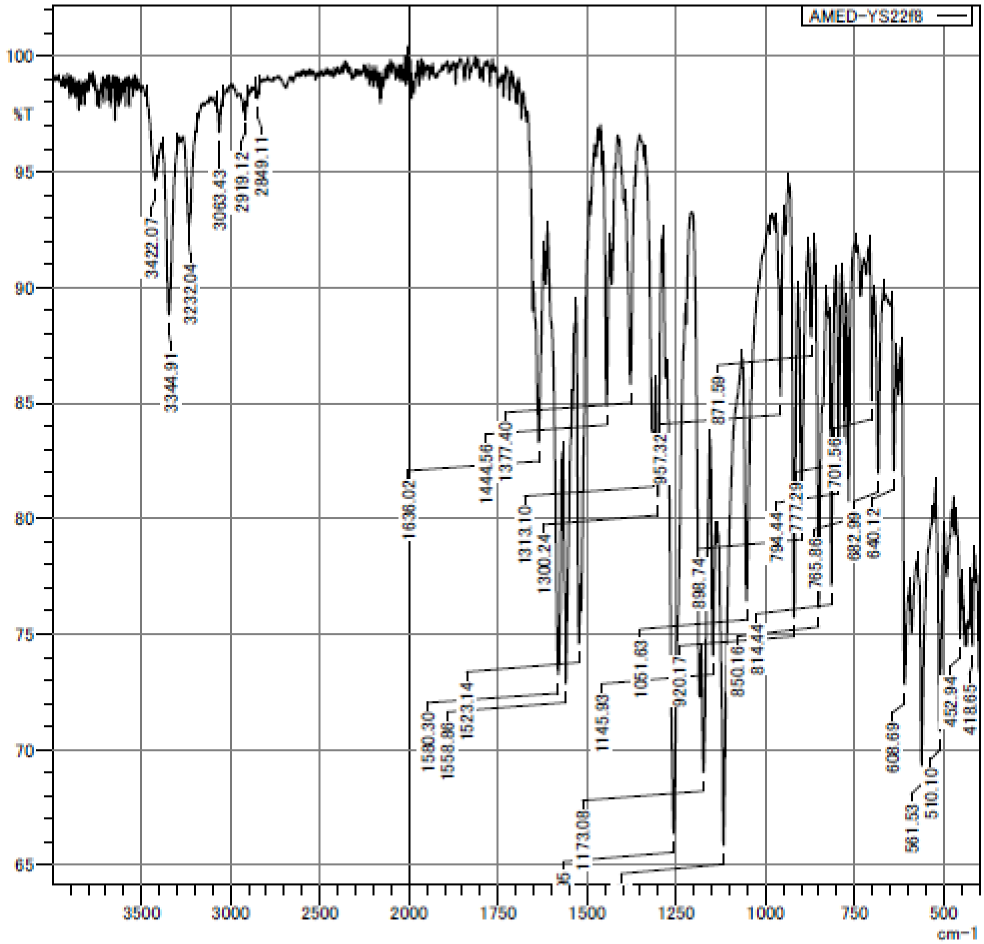

Figure S28. UV-VIS spectrum of compound **8** ($MeCN/H_2O$, 1:1)

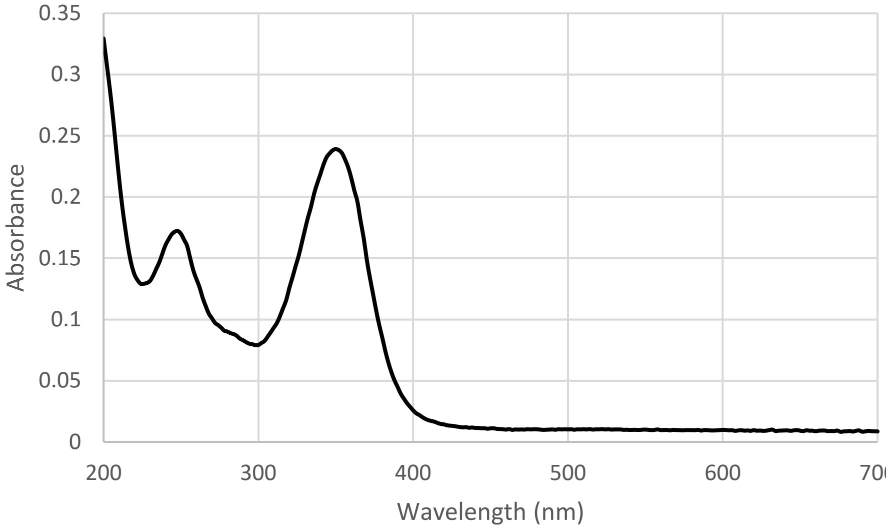

Figure S29. ^{1}H NMR spectrum of compound **9** (600 MHz, in $CDCl_3$)

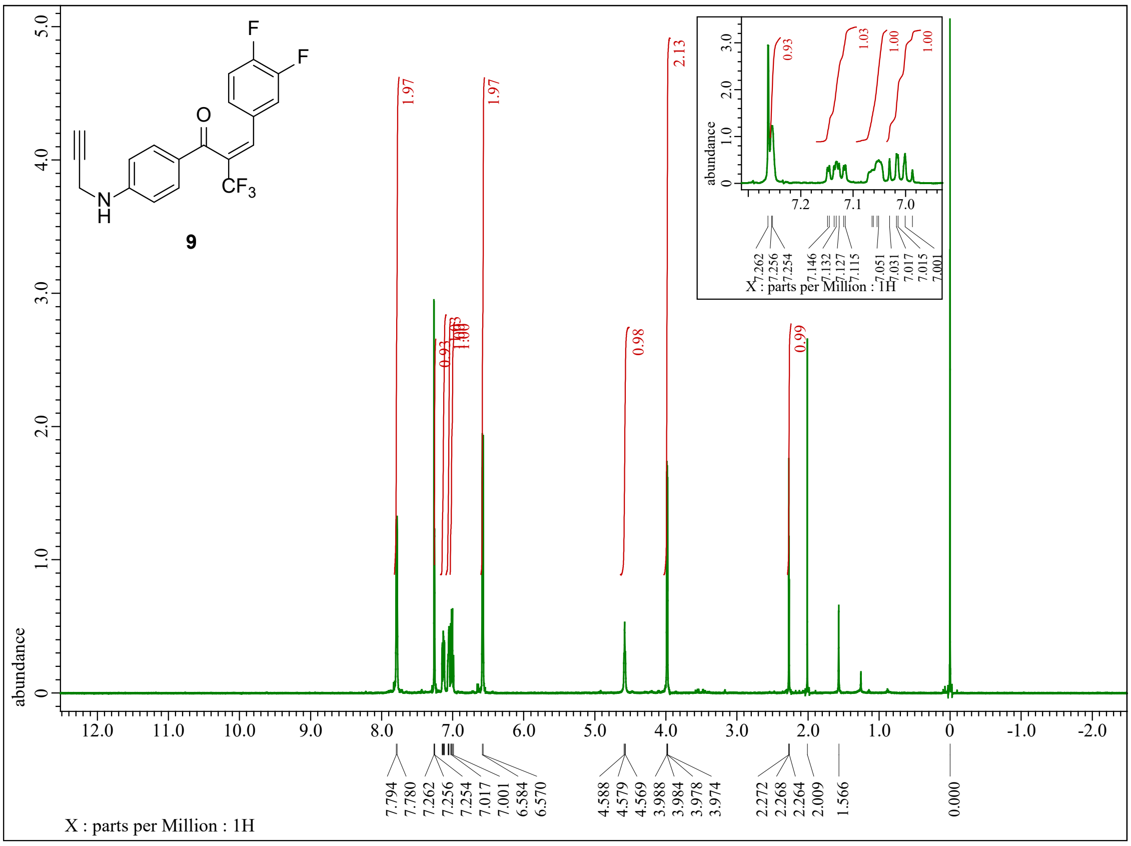

Figure S30. ^{13}C NMR spectrum of compound **9** (150 MHz, in $CDCl_3$)

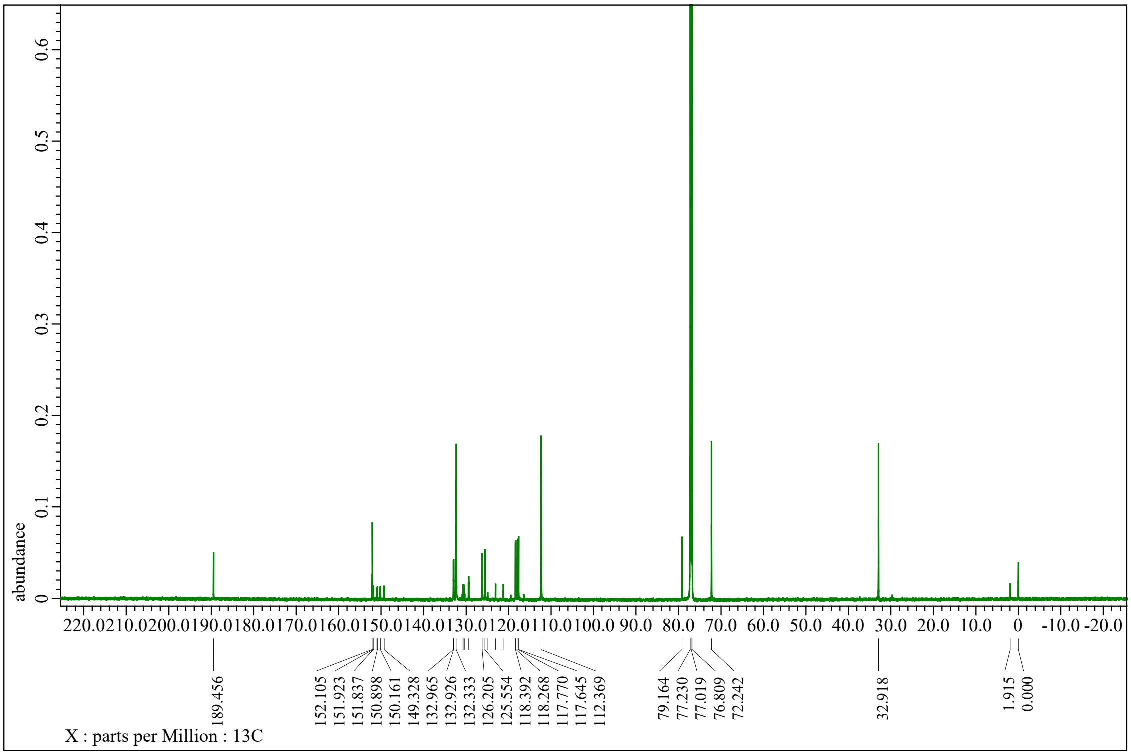

Figure S31. FT-IR spectrum of compound **9** (neat)

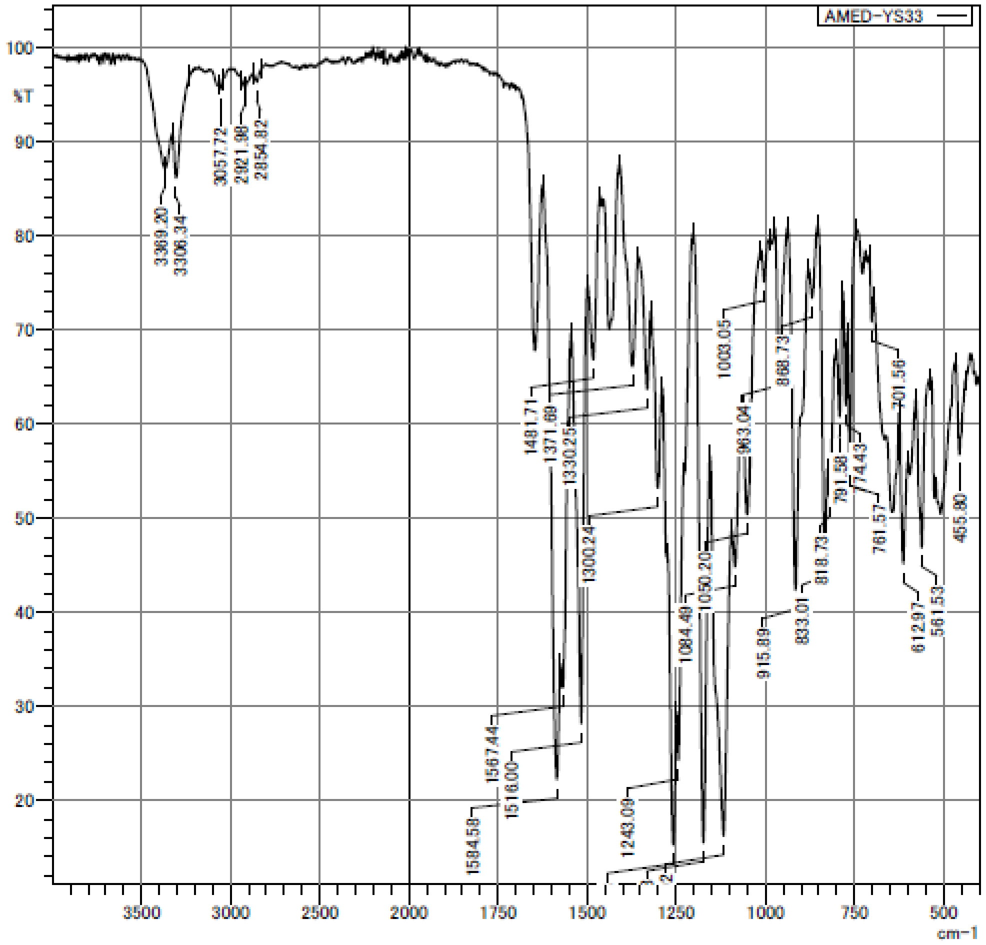

Figure S32. UV-VIS spectrum of compound **9** (MeCN/H_2O, 1:1)

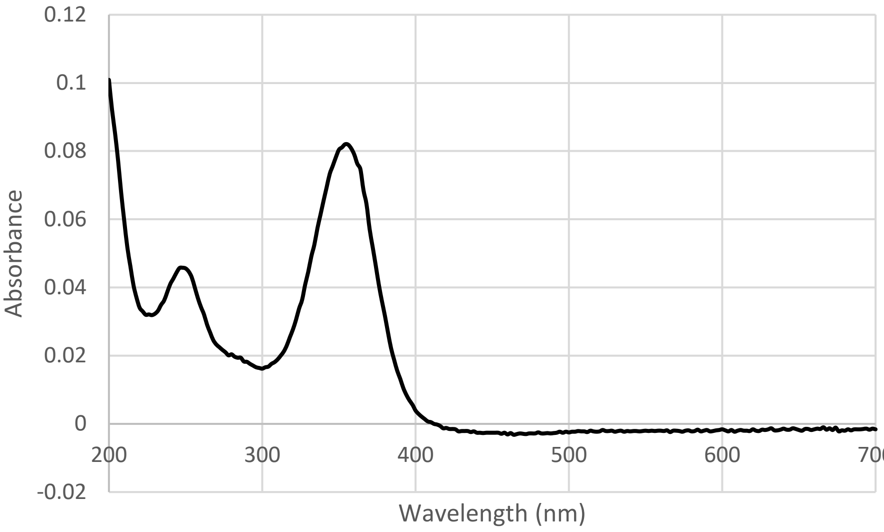

Figure S33. ^{1}H NMR spectrum of compound **10** (600 MHz, in $CDCl_3$)

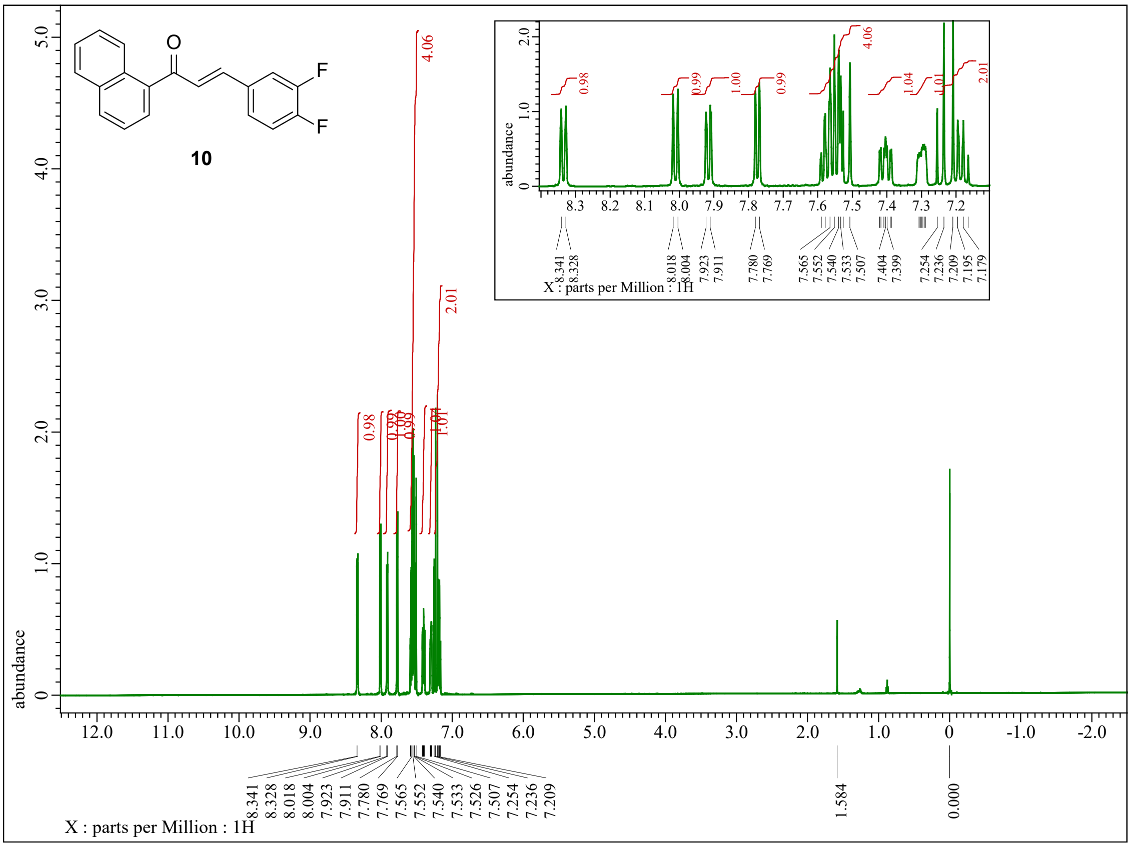

Figure S34. ^{13}C NMR spectrum of compound **10** (150 MHz, in $CDCl_3$)

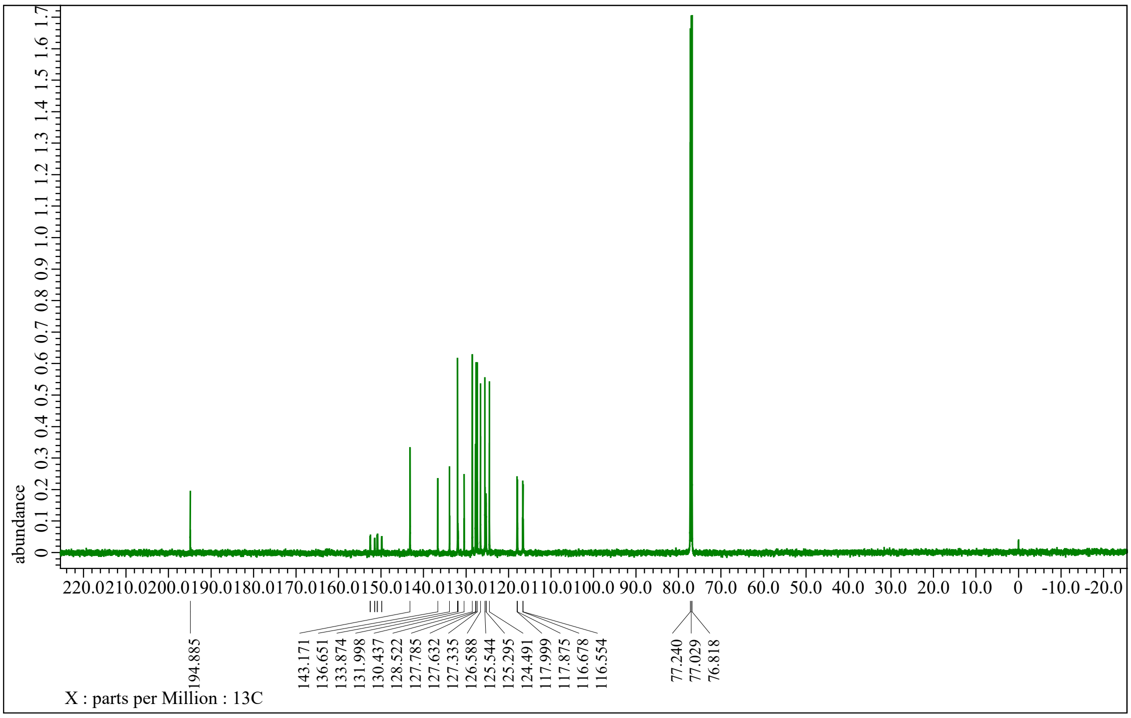

Figure S35. FT-IR spectrum of compound **10** (neat)

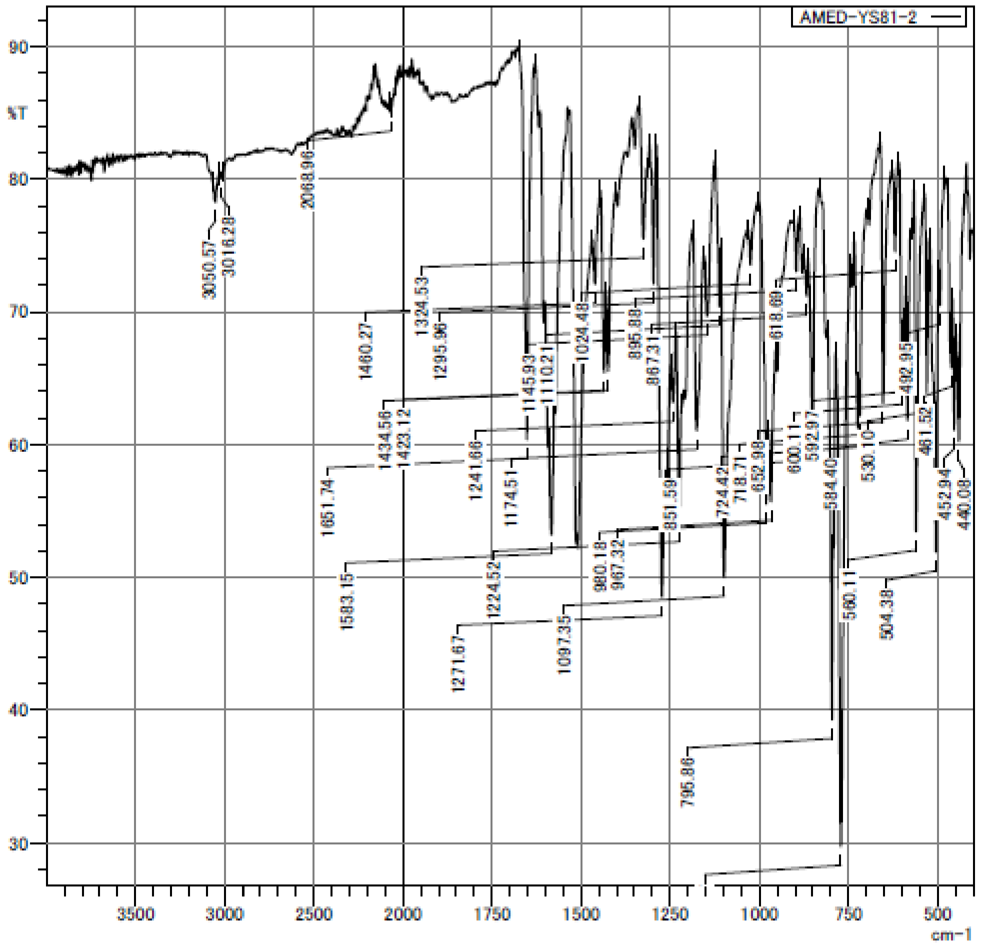

Figure S36. UV-VIS spectrum of compound **10** (MeCN/H_2O, 1:1)

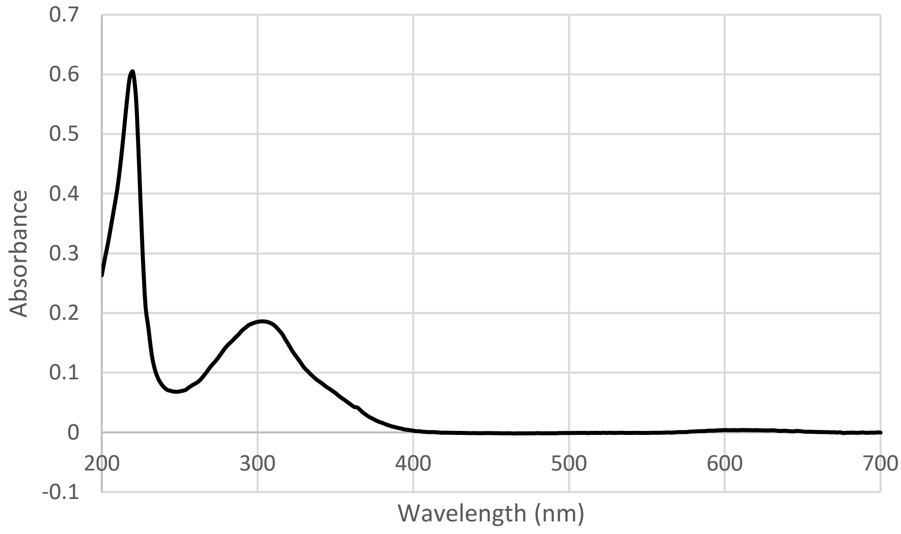

Figure S37. ^{1}H NMR spectrum of compound **11** (600 MHz, in $CDCl_3$)

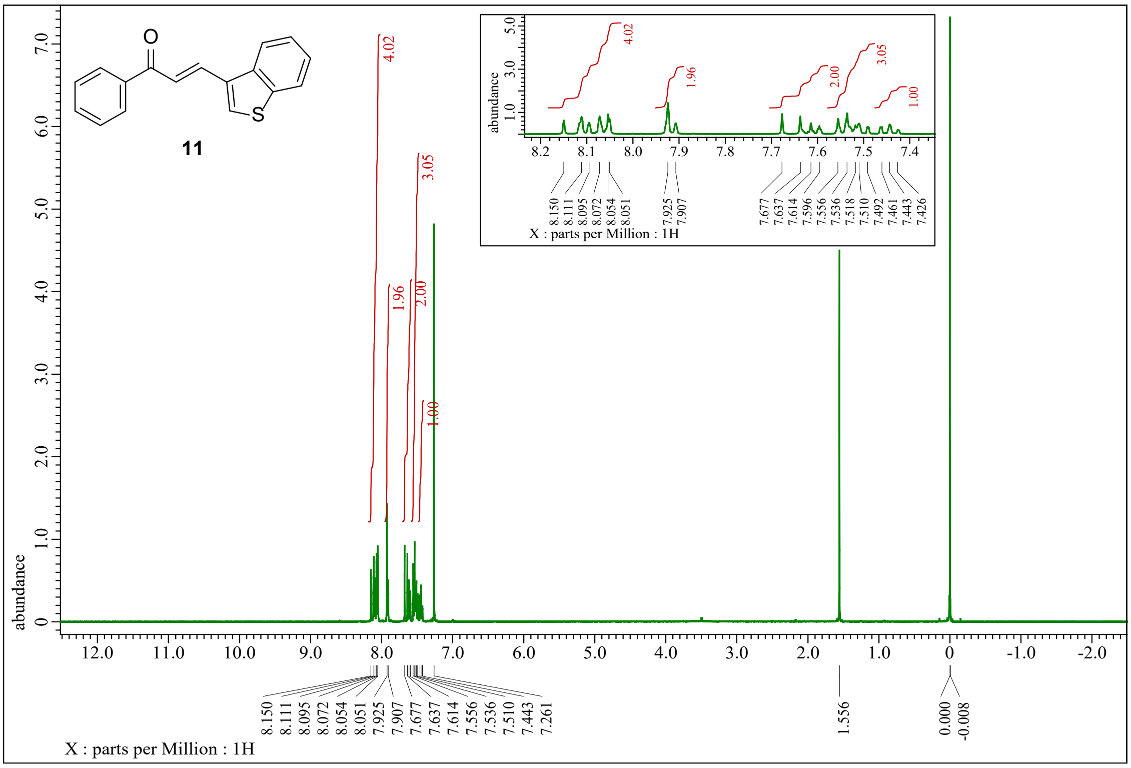

Figure S38. ^{13}C NMR spectrum of compound **11** (150 MHz, in $CDCl_3$)

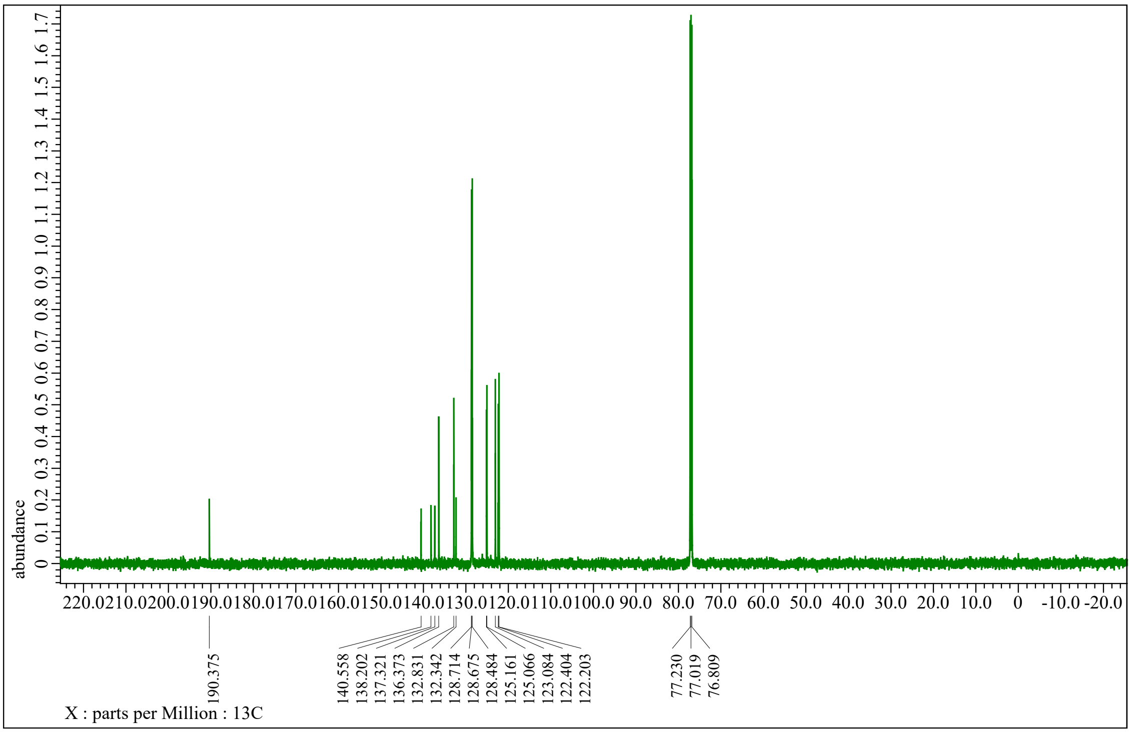

Figure S39. FT-IR spectrum of compound **11** (neat)

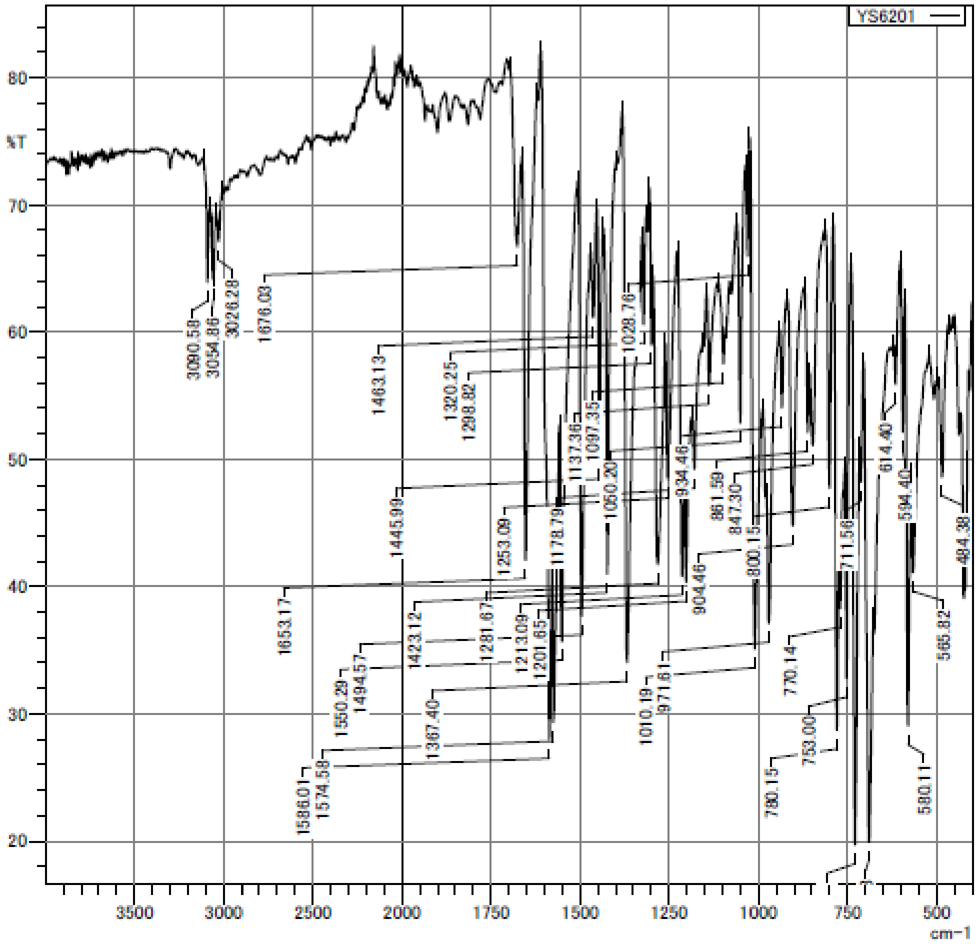

Figure S40. UV-VIS spectrum of compound **11** (MeCN/H_2O, 1:1)

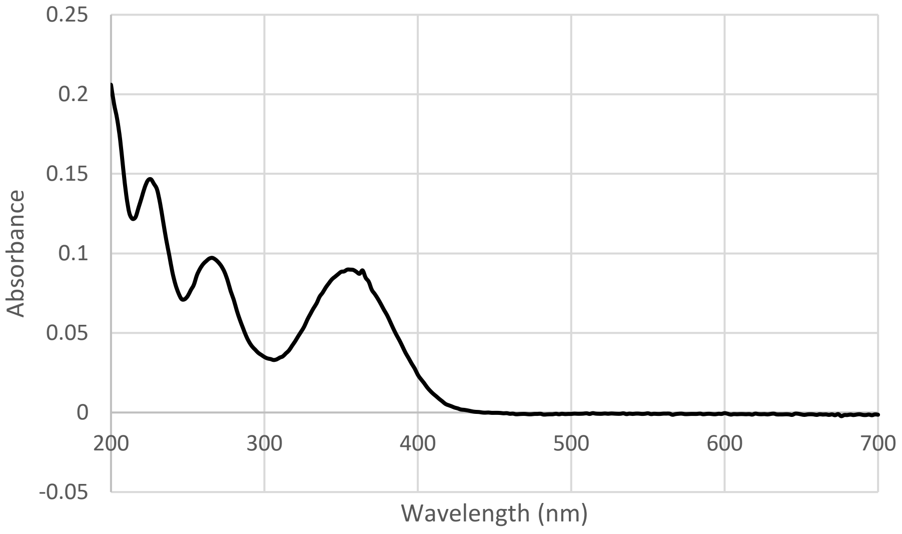

Table S1. Detailed information of deteced binding proteins.

Band No.	Accession	Description	Coverage [%]	# Peptides	# PSMs	# Unique Peptides	MW {kDa}
1	P08238	Heat shock protein HSP 90-beta	64	53	508	32	83.2
	P07900	Heat shock protein HSP 90-alpha	61	51	476	32	84.6
	Q16891	MICOS complex subunit MIC60	42	42	85	39	83.6
2	P11142	Heat shock cognate 71 kDa protein	52	35	201	28	70.9
	P38646	Stress-70 protein, mitochondrial	46	38	91	37	73.6
	A0A0G2JIW1	Heat shock 70 kDa protein 1B	37	28	107	16	70.1
3	P10809	60 kDa heat shock protein, mitochondrial	59	38	178	38	61
	P14618	Pyruvate kinase PKM	60	27	81	14	57.9
	P48643	T-complex protein 1 subunit epsilon	44	28	62	27	59.6
	P17987	T-complex protein 1 subunit alpha	50	25	63	25	60.3
	P50990	T-complex protein 1 subunit theta	41	23	43	23	59.6
4	P06733	Alpha-enolase	58	37	161	36	47.1
5	P60709	Actin, cytoplasmic 1	52	18	483	5	41.7
	P68032	Actin, alpha cardiac muscle 1	52	14	263	4	42
	Q9UQ80	Proliferation-associated protein 2G4	39	18	58	18	43.8
